# Application of head-mounted display-based augmented and mixed reality in nursing education: a scoping review

**DOI:** 10.1186/s12912-025-03413-1

**Published:** 2025-09-02

**Authors:** Ruifu Kang, Bohan Zhang, Shuojin Fu, Ling Tong, Shuai Jin, Yanling Wang, Qian Xiao

**Affiliations:** 1 School of Nursing, Capital Medical University, No.10 Xi-tou-tiao,You-an-men Wai, Feng-tai District, Beijing, China; 2 Centre for Smart Health, School of Nursing, The Hong Kong Polytechnic University, Kowloon, Hong Kong China

**Keywords:** Head-mounted display, Nursing education, Augmented reality, Mixed reality, Simulation, Scoping review

## Abstract

**Background:**

With Generation Z becoming the primary group of nursing learners and the increasing shortage of nursing education resources, augmented reality and mixed reality based on head-mounted displays are being used more and more in nursing education. However, the current application landscape and the proper usage of these concepts remain unclear. Therefore, this study aims to conduct a scoping review to explore the current applications of head-mounted display-based augmented reality and mixed reality in nursing education and to clarify the definitions and usage of the concepts of augmented reality and mixed reality, ultimately providing directions for future applications and research.

**Methods:**

Based on the five-stage framework and PRISMA-ScR guidelines, a comprehensive collection and summarization of evidence regarding the application of head-mounted display-based augmented reality and mixed reality in nursing education were conducted. The databases retrieved include CNKI, Wanfang Database, VIP, CBM, PubMed, Cochrane Library, Embase, CINAHL, Web of Science, Scopus, IEEE Xplore, ACM Digital Library, and Ei. The languages of the included literature are Chinese and English. The retrieval was up to October 2024.

**Results:**

A total of 44 studies were included in this review, covering three types of head-mounted displays: immersive head-mounted displays, smart glasses, and smartphone-based head-mounted displays. The main application areas were skills training and knowledge acquisition. Most of the studies were feasibility studies, though they also included some efficacy studies and research on personal experiences. In addition, these studies often employed vague or inconsistent definitions of augmented reality and mixed reality.

**Conclusions:**

Despite various explorations in the application of head-mounted display-based augmented reality and mixed reality in nursing education, there is still room for improvement in the relevant theory and utilization of this technology. In the future, interventions should use the three dimensions (observation of reality, real - virtual interaction, and fidelity of virtuality) described in detail, rather than simply employing the concepts of augmented reality or mixed reality. Efforts should be concentrated on developing and implementing head-mounted displays combined with other technologies that boast enhanced performance and cost - effectiveness, and further validating their effectiveness.

**Supplementary Information:**

The online version contains supplementary material available at 10.1186/s12912-025-03413-1.

## Background

### Nursing education needs new technologies

Generation Z who were born in the digital age, present a preference for integrating advanced technologies into nursing education. They prefer experiential learning methods over conventional lecture-based instruction and favor technological interactions over traditional textual materials [1]. At present, Generation Z has gradually become the main group receiving nursing education. A study has shown that 89.3% of students hold a positive attitude toward the use of technology in nursing education [2]. At the same time, the problem of insufficient human resources in nursing education also needs to be urgently addressed [3, 4]. Some studies have demonstrated that technologies such as virtual simulation, online learning, and gamified learning can enhance nursing students’ clinical competence and the efficiency of knowledge acquisition, boost their learning motivation and engagement, and reduce the demand for teaching personnel [5–7]. These findings collectively underscore the need to incorporate diverse advanced technologies to effectively complement conventional nursing education methods [8]. Among many technologies, immersive technology is an important application field in nursing education.

### Extended reality in nursing education

Immersive technologies based on constructivist theory and experiential learning offer a novel teaching approach that promotes active learning through simulated environments, hands-on practice, and innovation [9]. Extended reality (XR), a key component of immersive technologies [10], encompasses virtual reality (VR), augmented reality (AR), and mixed reality (MR), each of which can enhance learning experience in distinct ways [11]. Among these technologies, VR has the earliest development and is the most widely used in nursing education [12]. Several studies have evaluated the various applications of VR within nursing education, indicating its efficacy in enhancing students’ knowledge and skills [13–15]. However, there are some limitations of VR, such as the absence of tactile feedback, and the possibility of motion sickness, that pose challenges to its widespread adoption [16]. As alternative variants of immersive technologies, AR and MR have the potential to overcome certain limitations of VR partially and provide an enhanced nursing educational experience.

### AR and MR can bridge the reality and virtuality

In the realm of interactive technology, the primary characteristic of AR and MR is that they merge virtuality and reality rather than replace real environments, thereby enhancing visualization practices and generating a more authentic interactive experience [17]. For instance, AR and MR allow learners to retain haptic feedback on simulation manikins while enabling them to visualize internal anatomical structures [18]. Furthermore, the use of AR and MR extends to traditional educational materials through QR code scanning, augmenting them with digital data [17]. In the current state where more advanced technologies such as brain-computer interfaces are imperfect, AR and MR can preserve the perception of the real world while providing virtual learning materials or environments, and they are associated with milder adverse reactions. This compensates for the shortcomings of VR technology, which could not provide tactile feedback in a fully virtual environment and caused significant adverse reactions [19, 20]. These advancements suggested a broader prospect for their application in future nursing education.

### Head-mounted displays are important media for AR and MR

The application of AR and MR in nursing education has been primarily categorized into three types: handheld display, head-mounted display (HMD), and stationary display [21]. Handheld displays mainly include mobile phones and tablets. While they carry certain benefits of AR technology, these devices also have constraints such as the requirement to be manually held and they render a less immersive experience [22]. Stationary displays are mainly projectors, which can provide an experience of integrating virtual and real without wearing or holding any device. However, this kind of technology is not mature enough [23]. Contrastingly, HMD-based AR and MR technologies are more developed, providing immersive and hands-free user experiences [24, 25]. Given the current technological advancements, this presents the most promising avenue for integrating HMD-based AR and MR into nursing education [19, 20, 26].

### Current gaps and research objectives

Due to the broad prospects for the application of HMD-based AR and MR in nursing education, some reviews have discussed their use in this field. However, such reviews either conflated VR with AR or isolated AR from MR. For instance, there are two studies that reviewed the application of VR and AR in nursing education [27, 28]. Two reviews about MR covered the effects of MR on nursing simulation and critical thinking [19, 20]. Two other reviews on AR explored the effectiveness of AR in promoting independent learning in nursing and the application of AR in critical care education [26, 29]. These approaches not only suggested to readers a closer kinship between AR and VR but also artificially segregated the similar concepts of AR and MR, which is counterproductive to the unified research and application of these technologies. Therefore, it is more appropriate to discuss HMD-based AR and MR as a single technological approach. However, there is no evidence of a comprehensive review examining the application of HMD-based AR and MR technologies within nursing research. Therefore, this scoping review aims to provide a comprehensive review to determine HMD-based AR and MR status of application within nursing education and to analyze the use of AR and MR concepts in nursing education to clarify them, as well as provide future directions for the development of HMD-based AR and MR’s broader and more informed implementation in nursing education.

## Methods

A scoping review systematically maps the existing literature to identify key concepts, types of evidence, and research gaps, offering a structured overview of the field. We adopted this approach due to the fragmented and interdisciplinary nature of research on the application of HMD-based AR and MR in nursing education [30]. This method enables the synthesis of diverse studies to clarify the current state of knowledge, identify emerging themes, and guide future research directions. In this scoping review, we adopted the Arksey and O’Malley methodological framework consisting of five stages: (1) identifying the research question; (2) identifying relevant studies; (3) study selection; (4) charting the data; and (5) collating, summarizing, and reporting the results [31, 32]. This study was also based on the reporting guidelines of the Preferred Reporting Items for Systematic Reviews and Meta-Analyses Extension for Scoping Reviews (PRIMSA-ScR) [33]. The completed PRISMA-ScR checklist can be found in Additional file 1.

### Stage 1: identifying the research question

There were two research questions in this scoping review: (1) what is the current status of the application of HMD-based AR and MR in nursing education? (2) How do existing studies define and utilize the concepts of AR and MR?

### Stage 2: identifying relevant studies

A comprehensive and systematic literature search was conducted, guided by the elements of population, concept, and context (PCC) [34]. To encompass all individuals receiving nursing education, the population was defined as nursing students, midwifery students, and clinical nurses. The concept was HMD-based AR and MR, and the context included educational activities conducted within hospitals and other healthcare institutions. A total of 13 databases were systematically searched from their inception to October 2024, including Chinese National Knowledge Infrastructure (CNKI), Wanfang Data, VIP Database, Chinese Biomedical Literature Database (CBM), PubMed, Cochrane Library, Embase, CINAHL, Web of Science, Scopus, IEEE Xplore, ACM Digital Library, and Engineering Village (Ei). The search terms were developed using subject headings (MeSH and Thesaurus) and free text keywords. Meanwhile, we screened all retrieved studies by reviewing their titles and abstracts to identify reviews focusing on the application of AR or MR in nursing education. We then manually extracted all the primary studies included in these reviews and combined them with the directly retrieved original studies for subsequent screening. The full electronic search strategies for each database are provided in Additional file 2.

### Stage 3: study selection

After identifying all potentially relevant original studies, we imported them into EndNote (Version 21.5) for automatic duplicate removal. Subsequently, the data were imported into Zotero (Version 6.0.13) for screening based on the inclusion and exclusion criteria. Two researchers screened the titles and abstracts for eligibility and retrieved the full texts of potentially eligible studies for further assessment. Studies for which the full text was not available were also excluded. In instances of disagreements, discussions were conducted to seek resolutions. If consensus could not be achieved, the third researcher would address any inconsistencies. The inclusion and exclusion criteria for this scoping review were as follows: inclusion criteria included (1) studies published in English or Chinese; (2) studies involving nursing students, midwifery students, or clinical nurses; (3) studies focusing on nursing education using HMD-based AR and MR instructional methods; (4) instructional formats involving the use of HMDs to superimpose virtual objects onto real-world items, such as paper materials or mannequins; and (5) primary research studies. Exclusion criteria included (1) books or book chapters, reviews, editorials, conceptual analyses, and case reports; (2) studies involving only technical development without educational application; (3) survey studies not accompanied by the actual implementation of HMD-based AR and MR; and (4) studies utilizing HMD-based AR and MR for patient health care in clinical settings rather than for educational purposes. The PRISMA flow diagram summarized the selection of studies. The flow chart was created using an online tool based on the R language [35].

### Stage 4: charting the data

Data charting for the review was adapted from the data extraction tool in the Joanna Briggs Institute (JBI) manual for conducting scoping reviews [34]. The data to be extracted covered aspects such as author/year, aims, country, study design, participants, interventions (including HMDs, hardware, software, and triggers), use of AR and MR concepts, outcome measures, key findings, application domain, and research setting. These entries are made into a table in Excel software (Version 16.93). During data extraction, two researchers independently reviewed each included study and completed the corresponding entries for each article in the data extraction table. Upon completion, the entries were cross-checked. In case of discrepancies, a third researcher reviewed the relevant study and mediated the differences to reach a consensus. This process ensured the completion of data extraction and charting.

### Stage 5: collating, summarizing, and reporting the results

After data charting, the information in the table were organized into a hierarchical narrative description. This primarily included basic study characteristics, characteristics of the study participants, intervention features, application domains, and the use of AR and MR concepts. In addition, some results were visualized using the “scatterplot3d” package in R language (Version 4.2.2) [36, 37]. Due to the exploratory nature of this scoping review, the quality assessment was not conducted [31].

## Results

### Literature search and selection

**Fig. 1:**
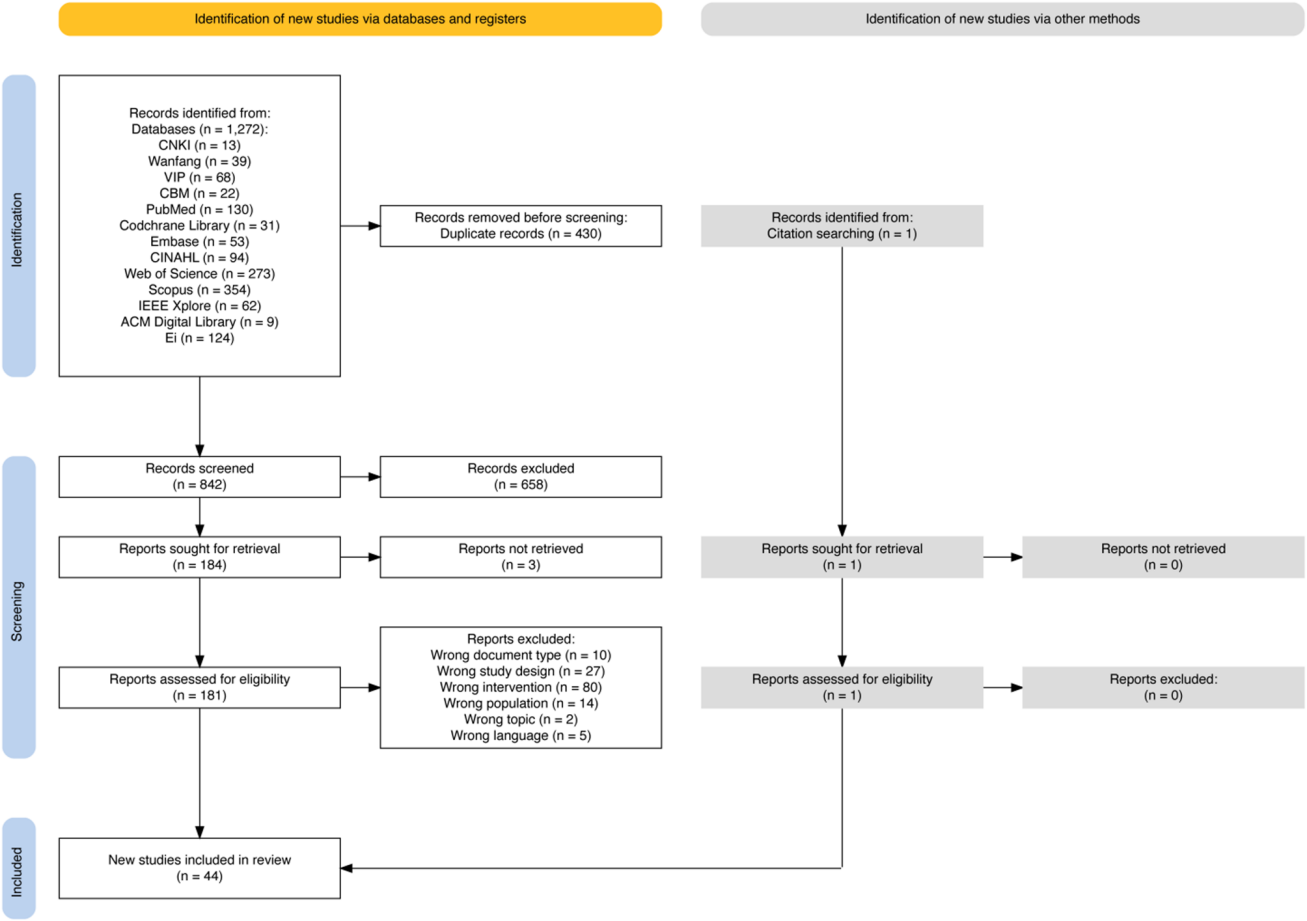
PRISMA flow diagram of the search and selection process

A total of 1272 studies were initially identified, and 430 duplicates were eliminated. After reviewing the titles and abstracts, 184 studies were included in the full-text screening, of which three were not found. After reading the full texts of the remaining 181 studies, 43 studies that met the research criteria were included in this study. In addition, we identified 80 original studies from eight reviews in this field. Among them, one study was not included in the original studies retrieved by our search and met the inclusion criteria, so we incorporated it as well. Finally, 44 studies were included for analysis (Fig. 1).

### Study characteristics

**Fig. 2:**
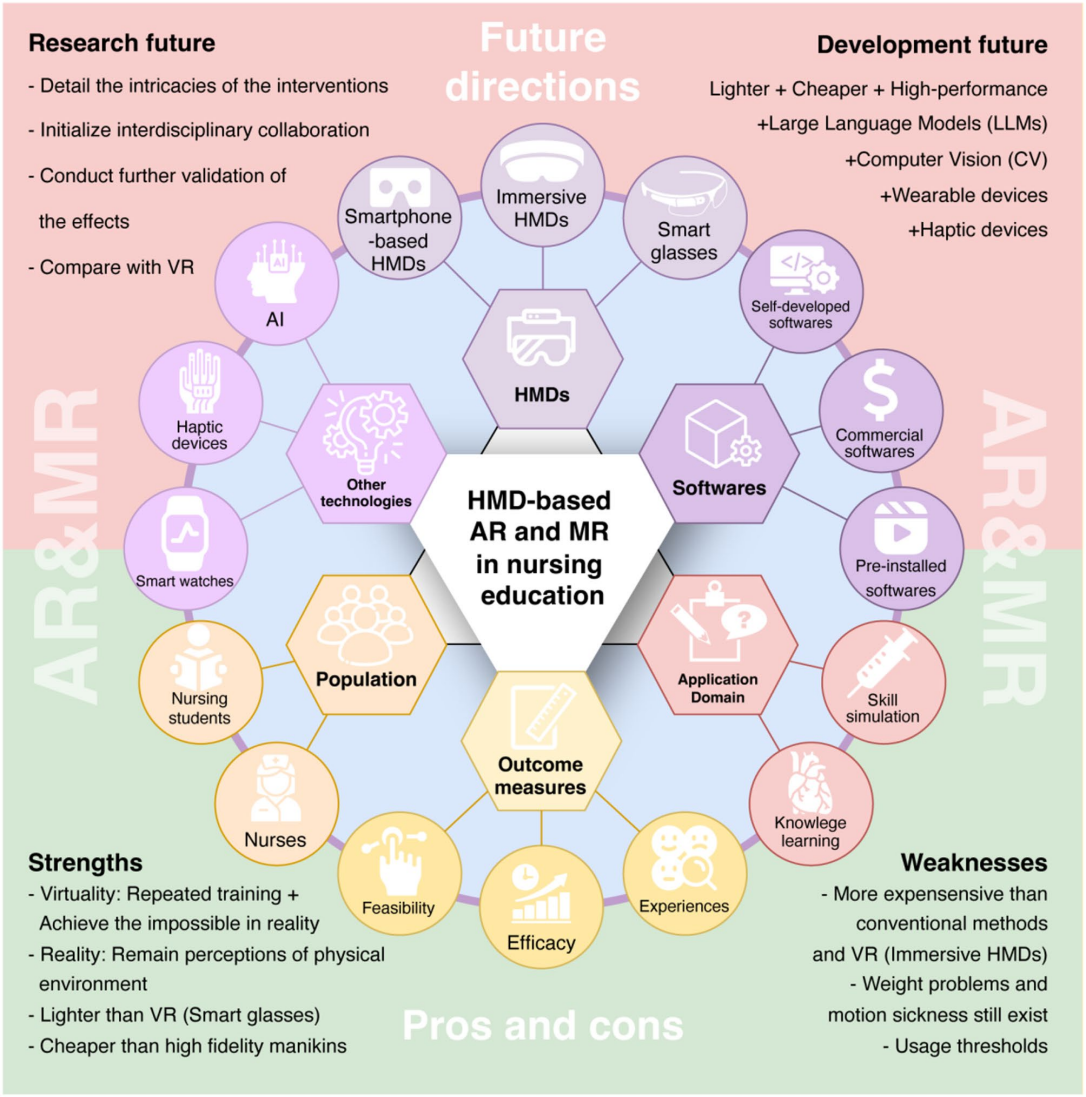
Result summary chart

Among these studies, 17 were from the United States [18, 38–53]; 12 were from South Korea [24, 54–64]; four were from China, with two in Mainland [25, 65], and two in Taiwan [66, 67]; three each from New Zealand [68–70], Australia [71–73], and Japan [74–76]; two from Germany [77, 78] (Fig. 2). The language was English and Chinese, and the publication year ranged from 2016 to 2024. In terms of research design, there were 21 mixed methods studies, seven quantitative randomized controlled trials, seven quantitative non-randomized controlled trials, five quantitative descriptive studies, and the remaining four were qualitative studies. In mixed methods research, the dominant design was commonly adopted, where quantitative research was the primary focus. There were 35 studies (80%) were conducted in universities, and nine studies (20%) were in hospitals. A summary of the 44 included studies is presented in Table 1. The status, advantages and disadvantages, and future directions of the application of HMD-based AR and MR in nursing education are summarized in Fig. 2.

**Table 1:** Desriptive characterics of included studies

Study	Country	Aims	Study Design	Measurements	Knowledge /Skill	Application domain	Setting
Vaughn, 2016	USA	Measure nursing students’ beliefs related to self-confidence and scenario design in simulated learning experiences when utilizing an innovative hybrid simulation to incorporate video technology using an augmented reality headset.	Quantitative descriptive study	1. Simulation Design Scale (SDS) 2. Self-Confidence in Learning scale (SCLS)	Skill	Acute asthma exacerbation scenario simulation	University
Barnett, 2017	Australia	Obtain user feedback from instructor and students on the application of a collaborative system that allowed students to undertake a clinical procedure with real-time audio and visual guidance provided by an instructor at a different location.	Mixed methods study	**Quantitative measurements:** 1. A 9-item survey about usability including ease of use, ease of learning, task satisfaction, copresence, and perception of engagement 2. The 5-item National Aeronautics and Space Administration Task Load Index (NASA-TLX) Mental Workload Rating Scale **Qualitative measurements:** Five open-ended questions	Skill	Dressing on the wound	University
Gruenerbl, 2018	Germany	Investigate the effect such a feed- back system can have beyond supporting immediate execution towards training the person to do it correctly in the future.	Quantitative non-randomized study	1. Chest compression (CC) depth 2. CC speed	Skill	Cardiopulmonary resuscitation (CPR)	University
Hauze, 2018	USA	Examine the use of immersive technology simulation delivered via the Microsoft HoloLens.	Quantitative randomized controlled trial	1. Instructional Materials Motivation Survey (IMMS) 2. SCLS 3. Knowledge measures	Skill	Learning Motivation: anaphylaxis	University
Lai, 2018	Taiwan, China	Develop a MR training prototype system for automated nursing skills training/exercise to achieve the goal of S-Learning by combining the related content knowledge including nursing skills testing process, designing of hybrid reality imaging system, motion sensing technology, 3D object modeling and dynamic performance, interactive situation design, and video content recognition technology.	Quantitative descriptive study	Only description	Skill	Nasogastric tube care	University
Hoyt, 2019	USA	Determine whether the use of a Virtual Standardized Patient, delivered via the Microsoft HoloLens, could improve the knowledge and skill of baccalaureate nursing students with regards to nursing assessment and intervention in a low- frequency, high-stakes scenario (anaphylaxis).	Quantitative randomized controlled trial	1. Knowledge and skill 2. Student satisfaction and self-confidence(SSSC)	Skill	Anaphylaxis care	University
Sugiura, 2019	Japan	Enhance the medical museum for more frequent usage and to make it more effective as a learning support environment, especially through self-directed learning using AR technology.	Mixed methods study	**Quantitative measurements**: A survey consisted of either yes or no to each of the three questions regarding usefulness, ease, and appearance **Qualitative measurements**: Free text comments	Knowledge	Anatomy learning	University
Balian, 2019	USA	Test the validity of augmented reality cardiopulmonary resuscitation training system against CPR guidelines and evaluated participants’ perception of this educational tool.	Mixed methods study	**Quantitative measurements**: 1. Mean CC rate in compressions per minute 2. Mean CC depth in millimeters 3. Percent of CCs with complete chest recoil **Qualitative measurements**: Open-ended questions about satisfication	Skill	CPR	University
Chen, 2019	Taiwan, China	Identify whether the integration of AR helped increasing learners’ learning Effectiveness and motivation.	Mixed methods study	**Quantitative measurements**: Questionaire about effectiveness and satisfaction **Qualitative measurements:** semi-structure interview	Knowledge	English for Specific Purpose (ESP)	University
Wunder, 2020	USA	1. Assess learners’ technical skills while managing a simulated fire using mixed reality. 2. Assess learners’ non-technical skills while managing a simulated fire using mixed reality.	Quantitative descriptive study	1. Technical Skills Scores 2. Non-Technical Skills Scores	Skill	An emergent fire during a simulated tracheostomy procedure	Hospital
Frost, 2020	Australia	Explore the contemporary application, inclusive of advantages and challenges of MR technology in the education of nursing students and, its contribution to enhanced learning.	Qualitative study	A 14-item questionnaire which included demographic information and open-ended questions which explored the participants learning experience using Microsoft Hololens, including their opinions with regards to the potential for enhancing learning	Skill	Anaphylaxis patient	University
Kim, 2020	Korea	Design and usability test of a smart glass application for XR assisted training of core nursing skills.	Mixed methods study	**Quantitative measurements**: 1. Convenience of application 2. The covenience of using the glass 3. Text readability 4. Are pictures understandable 5. Understanding the skills **Qualitative measurements**: What is your overall feeling for our application? Have you encountered any difficulties during the experiment?	Skill	Blood transfusion and intradermal injection	University
Leary, 2020	USA	Examine the use of an AR CPR training application compared with a standard CPR training manikin to determine if the AR technology could improve CPR quality defined as chest compression rate and depth.	Quantitative randomized controlled trial	1. CC depth 2. CC rate 3. CC fraction 4. Self-developed questions: feeling	Skill	CPR	Hospital
Frost, 2020	Australia	1. Explore the perceptions of need that health professional students from different disciplines formulated after a visual assessment of the same patient using MR. 2. Explore the recognition of a potentially life-threatening situation by different health care students.	Qualitative study	Perception of patient’s needs	Skill	Interprofessional collaborative practice about myocardial infarction	University
Kopetz, 2020	Germany	1. Develop a smart glasses support for skills training with User-Centered Design. 2. Evaluate the feasibility of this prototype.	Quantitative descriptive study	1. Number of errors and time on task 2. Self evaluation of their performance 3. Confidence and comfort 4. Participants’ familiarity with the task and how well-suited they found the smart glasses support in training and on the job 5. Usability assessment with an adapted Post-Study Usability Questionnaire (PSSUQ)	Skill	Skills about transferring the patient into the wheelchair	University
Anderson, 2021	USA	Answer the following questions: 1. How did participants feel about the usability of the triage scenario in AR? 2. Did participants find the triage simulation effective in AR?	Mixed methods study	**Quantitative measurement**: 1. System Usability Scale (SUS) 2. Simulation Effectiveness Tool-Modified (SET-M) **Qualitative measurements**: Open-ended questions	Skill	Adult-gerontology acute care	University
Kim, 2021	Korea	Test the feasibility and usability of implementing a core nursing skill training program that combined visualization and XR technology for undergraduate nursing students.	Mixed methods study	**Quantitative measurements**: 1. Seventeen items usability test 2. Satisfaction test 3. Observational **Qualitative measurements**: Qualitative questions about experiences	Skill	Blood transfusion and intradermal and injection administration	University
Collins, 2021	New Zealand	Evaluate the efficacy of using standardized holographic patients to assist undergraduate nurses to develop clinical reasoning skills thereby improving quality and safety of patient care.	Mixed methods study	**Quantitative measurements**: Virtual Patient Version of the Lasater Clinical JudgementRubric **Qualitative measurements**: Satisfication, benefits, disvantages	Skill	Clinical reasoning skills training	University
Yoon, 2021	Korea	Assess the feasibility of a desktop user interface to monitor remote collaboration systems using the latest Google Glass (Glass EE 2) and to determine whether real-time video and audio provided via Glass EE2 is helpful, informative, and provides adequate information needed in emergency care settings.	Qualitative study	A structured self-developed questionnaire	Skil	Cooperative training about arrhythmia nursing	University
Bektic, 2021	USA	1. Develop a programmable platform that simulates deformable edema levels using AR glasses combined with a stylus haptic device. 2. Examine the efficacy of the simulation system by conducting a user study with human subjects.	Mixed methods study	**Quantitative measurements**: A Task Load Index chart **Qualitative measurements**: The long form questions	Skill	Edema assessment	University
Toto, 2021	USA	1. Determine the feasibility of collecting data on the timing and volume of fluid administrated during septic shock simulation with and without the use of PediSepsisAR. 2. Describe PediSepsisAR as an educational tool in septic shock simulation.	Quantitative randomized controlled trial	1. Time to administer 2. Facilitators timed how long participants took to verbalize they had recognized	Skill	Fluid administration during pediatric septic shock simulation	Hospital
Dias, 2021	USA	1. Convert a standard disposable direct laryngoscope into a video laryngoscope. 2. Investigate whether AR improved novice intubation proficiency on manikins compared to traditional methods.	Quantitative randomized controlled trial	A series of metrics related to the success rate and time required for neonatal intubation.	Skill	Neonatal intubation training	Hospital
Lee, 2021	Korea	Examine whether adaptation to smart glasses would produce beneficial effects on learning outcomes with team-based simulation by improving team dynamics and learning engagement.	Mixed methods study	**Quantitative measurements**: 1. Usability questionnaire 2. The Attitudes Towards Interprofessional Health Care Teams Scale 3. Learning satisfaction **Qualitative measurements**: Essay questionnaire	Skill	Team-Based Simulations for Emergency Scenarios	University
Zhang, 2021	China	Explore the effect of AR technology combined with immersive experiential lectures in the practical teaching of vascular surgery nursing.	Quantitative non-randomized study	1. Theoretical Knowledge 2. Operational Techniques 3. Diagnostic and Therapeutic Technology 4. Medical Record Writing 5. Teaching satisfaction	Skill	Vascular Surgery Nursing	Hospital
Anderson, 2022	USA	Evaluate the following indicators: 1. Amount of time for participants to complete the AR orientation and AR prebrief scenario, that is, activity. 2. Usability and effectiveness of the AR activity during the prebrief phase. 3. Experienced side effects from the AR activity during the prebrief phase. 4. Effectiveness of the high-technology, manikin-based simulation following the AR prebrief activity.	Mixed methods study	**Quantitative measurements**: 1. SUS 2. Virtual Reality Sickness Questionnaire (VRSQ) 3. Time 4. SET-M **Qualitative measurements**: Open-Ended Questions	Skill	AcuteCare	University
Adams, 2022	New Zealand	Consider the impact of using MR in nursing education and in particular anatomy studies.	Quantitative descriptive study	Learning Object Evaluation Scale for Students	Knowledge	Anatomy learning	University
Kim, 2022	USA	Investigate the impact of AR on undergraduate students’ capacity to learn human anatomy compared to traditional methods.	Mixed methods study	**Quantitative measurement**: A 5-point Likert scaleto measure various dimensions of cybersickness (affective and physical) **Qualitative measurements:** Questions about academic application, affective experience, and physical experience.	Knowledge	Anatomy learning	University
Kang, 2022	Korea	Develop and implement MR-based online interprofessional education (IPE) in which medical and nursing students could engage and develop interprofessional understandings.	Quantitative non-randomized study	Satisfaction with Simulation Experience Scale (SSES)	Skill	IPE about ischemic stroke	University
Qi, 2022	New Zealand	Approach a teaching platform to enable students to study synchronous in the classroom and at home.	Mixed methods study	**Quantitative measurements**: Pre-post knowledge test **Qualitative measurements**: Interveiew	Skill	Knowledge revision	University
Heo, 2022	Korea	1. Determine the effectiveness and feasibility of AR-based learning for novices to set up a ventilator by focusing on independently completing the procedures and assessing the degree of assistance required. 2. Evaluate the step characteristics in terms of the precision and assistance required.	Quantitative randomized controlled trial	1. Overall score of the procedure 2. The required level of assistance 3. Short questions on 3 themes: confidence, suitability, and whether they intended to recommend AR system to others SUS	Skill	Mechanical Ventilator Setup	Hospital
Menon, 2022	USA	1. Conduct pilot study to determine the feasibility and acceptability of using AR in nursing education. 2. Evaluate performance of AR group in comparison with control group in the pilot study.	Quantitative non-randomized study	1. The Student Satisfaction and Self-Confidence in Learning 2. A researcher-developed rubric was used to measure students’ physical assessment performance-based	Skill	Physical assessment of lung/heart	University
Stelter, 2023	USA	Illuminate the expected and unanticipated early implementation challenges of integrating AR technologies into anatomy labs.	Mixed methods study	**Quantitative measurements**: Nine questions about usage of AR in anatomy lab courses **Qualitative measurements**: Open ended questions	Knowledge	Anatomy learning	University
Kim, 2023	Korea	Develop a human anatomy-based skill training system and pilot test its usability and feasibility.	Mixed methods study	**Quantitative measurements**: 1. Usability test 2. Learning satisfication 3. Competency **Qualitative measurements**: User feedback	Knowledge	Anatomy learning	University
Nakazawa, 2023	Japan	1. Evaluate the effectiveness of AR for affective communication training for caregivers. 2. Investigate the relationship between the training results and participants’ personalities.	Quantitative randomized controlled trial	1. Shortened version of the Japanese Big-Five Scale 2. Jefferson Scale of Empathy Health Profession Students’ version 3. Face-to-face distance and pose 4. The occurrence of eye contact between the participants and simulated patients 5. Length of the caregiver’s speech	Skill	Communication skill	University
Son, 2023	Korea	Provide basic data about nursing students’ perception and experience of MR-based education to promote its effective application in the field of nursing education.	Qualitative study	Semistructured interview: What have you experienced using the HoloPatient? What were the advantages of using the HoloPatient for COVID-19 case studies? What were the challenges of or concerns about using the HoloPatient? What needs to be improved?	Skill	Covid-19 simulation	University
Kleinman, 2023	USA	Determine the impact AR-CPR has on CC performance in community emergency department non–pediatric specialist providers and to identify themes around user feedback on the AR-CPR system to inform iterative improvements.	Mixed methods study	**Quantitative measurements: ** 1. The peroptimal rate of CCs (100–120 compressions per minute)centage of 1-min epochs a participant was at the 2. CC depth of 5 cm (2.54–5.72 cm; representing upper and lower limits of the user interface) 3. Combination of optimal rate and depth (defined as goal CC) 4. Retention of CC performance after removal of AR-CPR and assessment of hardware and software issues **Qualitative** **measurements: ** semi-structured interview about user experience	Skill	CPR	Hospital
Liu, 2023	China	Compare the efficacy and satisfaction of mixed reality technology and conventional methods in case-based teaching of intracranial aneurysm care.	Quantitative non-randomized study	1. Knowledge test 2. Satisfication	Skill	Intracranial aneurysm nursing	Hospital
Woo, 2023	USA	1. Determine what visual variable factors most influence the perception of difficulty for needle insertion through text and image-based surveys. 2. Combine the visual variability in mixed reality with a virtual training system with haptic feedback, allowing nursing students to train with variability.	Mixed methods study	**Quantitative measurements**: 1. Time 2. Angle 3. 3D motion data 4. The time participants took to insert the needle into the vein (1 attempt) 5. The time to complete each trial (4 attempts). **Qualitative measurements:** 1. The needle insertion state 2. The hand model type, 3. The data from the NASA TLX form	Skill	Needle Insertion Simulation	University
Kim, 2023	USA	Develop a system for nursing students or healthcare professionals to practiceIntravenous Injection needle insertion into a virtual arm with unlimited attempts under various changing insertion conditionsand test its usability.	Mixed methods study	**Quantitative measurements**: 1. Success rates of needle insertion 2. The insertion angles (5 to 30 degrees) 3. Task completion time (start and end) 4. Distance (the needle tip end to the vein center) **Qualitative: measurements: ** ubjective responses to both the questionnaire and NASA TLX	Skill	Needle Insertion simulation	University
Kang, 2023	Korea	Evaluate the effect of Problem-based learning supported by MR on perception of problem-solving ability, critical thinking disposition, and learning confidence and satisfaction of nursing students in South Korea.	Mixed methods study	**Quantitative measurements: ** 1. Perception of problem-solving ability 2. Critical thinking disposition 3. Learning confidence and satisfaction with HoloPatient-based PBL **Qualitative** **measureme** **nts: ** The interviews with two focus groups to explore nursing students’ perceptions of HoloPatient-based PBL experience	Skill	Solving nursing problems in older adult withdelirium or delirium superimposed on dementia admitted to the emergency department	University
Choi, 2024	Korea	Develop and implement team-based emergency nursing simulations for clinical nurses using MR, sscertain the effects on critical thinking, clarity of communication, communication confidence, learning transfer motivation, and immersion in simulation during emergencies.	Quantitative non-randomized study	1. Critical thinking 2. Motivation of learning transfer 3. Communication clarity 4. Communication confidence 5. Learning immersion in simulation	Skill	CPR	Hospital
Moon, 2024	Korea	Evaluate the effectiveness of integrating MR preparation into simulation education, focusing on enhancements in knowledge, self-confidence in learning, and self-efficacy in learning. Additionally, they aimed to assess the usability of MR as a preparatory tool in simulation training, specifically examining formative evaluations of performance, practice immersion, and satisfaction.	Quantitative non-randomized study	1. Knowledge 2. Self-confidence in learning 3. Self-efficacy in learning 4. Satisfaction 5. Practice immersion 6. Group performance	Skill	Acute coronary syndrome	Hospital
Arakida, 2024	Japan	Compare the learning outcomes of the AR tool with traditional training mannequins and identify potential uses and improvements of the AR tool.	Mixed methods study	1. Understanding and interest in the AR tool 2. Results of skill tests 3. Amount of time required for endotracheal aspiration in the skill test	Skill	Endotracheal aspiration	University
Yoo, 2024	Korea	Develop, introduce, and evaluate an AR-based educational program designed for nurses, focusing on its potential to facilitate hands-on practice and self-directed learning.	Mixed methods study	1. Technology acceptance 2. Usability test	Skill	Extracorporeal Membrane Oxygenation (ECMO) using	Hospital

### Characteristics of subjects

A total of 2410 individuals were included in this study. There were 35 studies involving nursing students, with 24 of these studies focused solely on nursing students and the remaining 11 included nursing students and faculty from other medical specialties. The sample sizes varied widely, ranging from a maximum of 232 to a minimum of just five participants. A total of nine studies featured clinical nurses as study subjects. Among these, five studies focused exclusively on nurses, while the other four also included other medical personnel (such as doctors and technicians). In these studies, the sample size ranged from 24 to 100. Details of the study subjects and interventions can be found in Additional file 3.

### Characteristics of interventions

#### Head-mounted displays

The HMDs involved in this study were primarily categorized into three types: immersive HMDs, smart glasses, and smartphone-based HMDs. There were 72% studies used immersive HMDs (32/44), which mainly included products like HoloLens, Magic Leap One, HTC Vive, and Vzix Wrap920AR [47, 58, 74, 76]. These HMDs boasted powerful performance, capable of overlaying realistic three-dimensional images onto real-world scenes, often without the need for triggers (such as QR codes or markers). They achieved this through environmental perception, enabling the placement of virtual objects at specific locations [72]. In current nursing education, three studies used immersive HMDs that allowed learners to recognize the impact of their maneuvers on patient anatomy and physiology while practicing and simultaneously enhanced their theoretical knowledge and operational proficiency [18, 46, 76]. However, immersive HMDs also faced issues such as dizziness and significant weight [40, 42]. Smart glasses, which comprised 23% (10/44) in this research, as another form of HMD, are lightweight but less immersive [59]. They typically only displayed flat-form videos, images, and presentations. Additionally, they featured video recording and voice communication capabilities [77]. HMDs based on smartphones generally used an HMD-shaped supporting shell into which the smartphone is inserted for relatively simple AR effects. Such HMDs were not lightweight and had mediocre display quality, However, they put commonly used smartphones in paper head-mounted brackets (such as Google Cardboard), thus providing strong convenience [67]. In this review, 5% of studies used HMDs based on smartphones (2/44).

#### Software

The software included in this review could be categorized into three main types. The first type was self-developed by the research teams, accounting for 50% (22/44) of the interventions. This approach was predominantly adopted by nursing education teams in collaboration with technical staff, to create customized software that was appropriate for specific teaching scenarios. The second type involved the use of existing commercial software, such as HoloPatient (10/44), CPReality (2/44), AnatomyX (2/44), AresAR (1/44) PediSepsisAR (1/44), and Microsoft Dynamics 365 (2/44). HoloPatient could project patients with various diseases as holographic images into the real world. CPReality combined simulation models for CPR skill training and evaluation [40, 46]. AnatomyX allowed nursing learners to interact directly with 3D anatomical models using their hands, enabling them to rotate, dissect, and other manipulations to learn anatomy [50, 51]. These commercial software products all used HoloLens as their main hardware platform, although HoloPatient also supported mobile platforms like smartphones. The third type of software was built into the HMDs, with studies focused on using the software built into the HMDs themselves, adding or linking pre-designed images and videos for playback and interaction, without the need for additional software development [41, 52]. Two other studies did not mention any software-related information [25, 65].

#### Hardware

Besides HMDs, some studies also integrated other smart hardware or sensors with HMDs to enhance interactivity. There are two studies that combine haptic feedback devices with HoloLens for injection skill training [44, 53]. Through haptic feedback devices, nursing students could perceive the pulse of a virtual patient, providing a more realistic experience for the virtual injection training scenario. Another study used smartwatch in conjunction with Google Glass for CPR training [77]. The sensors on the chest of the simulation model could send information such as the speed and frequency of the students’ compressions to the smartwatch and Google Glass. The smartwatch was used as a complement to the visual and auditory feedback provided by Google Glass, providing haptic prompts through vibrations, adding visual information, and achieving multisensory feedback for CPR training. There are also three studies that use sensors (such as rheostats and barometers) to map learners’ operations to virtual models, enabling skill learning to have real feedback [18, 45, 74].

### Application domains

#### Skill training

Skill training and simulation were the main application scenario of HMD-based AR and MR in nursing education, accounting for 86% (38/44) of the instructional fields in this study. This included CPR training [40, 77], COVID-19 simulation [62], interdisciplinary training [57, 73], communication skills training [75], emergency scenario simulation [38, 52], and other individual skill training [44, 67]. Applying HMD-based AR and MR in CPR skill training allowed learners to deepen their theoretical understanding of the skills by experiencing tactile feedback and observing the impact of CPR maneuvers on the circulatory system on simulation models [40, 46]. Three studies applied HMD-based AR and MR technologies for interdisciplinary skill training to compare and train students from different specialties in the assessment of virtual patients [57, 60, 73]. Students and professionals from various medical specialties collaborated and learned through the voice and recording features of smart glasses, thereby enhancing their individual skills and interdisciplinary collaboration [57].

#### Knowledge learning

Theoretical knowledge learning was another application scenario for HMD-based AR and MR in nursing education, accounting for 14% (6/44) of this study, which was relatively lower compared to skill learning. Among these, five studies focused on the learning of anatomical knowledge [50, 51, 58, 68, 76], and one on the learning of professional nursing English [66]. HMD-based AR and MR could provide natural interaction and enhance the 3D models of anatomy, which could increase the learning interest and motivation of students [68]. Through HMD-based AR and MR, the English names of corresponding nursing supplies could be viewed, and it was shown that active learning was promoted among nursing students [66].

### Outcome measures

#### Feasibility

Feasibility indicators included usability [39], acceptance [63], learning confidence [43], learning motivation [42], and learner satisfaction [58], were collected mainly through surveys, open-ended questions, and interviews. Research tools were variable and some studies developed their own tools [48, 64]. However, some existing well-established tools were used, such as the System Usability Scale (SUS), which was used to assess the usability of HMD-based AR and MR [38, 55]. Some studies utilized the Self-Confidence in Learning Scale (SCLS) to evaluate learners’ confidence [42, 52], and the Instructional Materials Motivation Survey (IMMS) to assess learning motivation [42]. Some tools which were originally developed and applied in VR contexts, have been adapted for HMD-based AR and MR research. For instance, the Virtual Reality Sickness Questionnaire (VRSQ) was used to investigate discomfort in participants using HMD-based AR and MR [38]. The National Aeronautics and Space Administration Task Load Index (NASA-TLX) was also used in several studies to measure the cognitive load of learners using HMD-based AR and MR [44, 47, 53, 71]. Overall, nursing students had a high level of satisfaction with HMD-based AR and MR, showing a preference for these new methods over traditional teaching approaches [51, 58]. However, HMD-based AR and MR also had some similar but less obvious drawbacks to VR devices, such as the high weight burden (immersive HMDs) and the occurrence of motion sickness [51]. In addition, there was research pointing out that the acceptance of HMD-based AR and MR in anatomy learning and the learning curve was related to the age of the learners [68].

#### Efficacy

The efficacy measures varied in studies, depending on the specific teaching domain covered. These measures were primarily divided into subjective and objective indicators. Subjective indicators were generally obtained through surveys completed by learners or observations made by researchers or teachers, such as designing knowledge test questions or nursing operation checklists in specific domains [25, 70]. Some studies used generic scales in simulation teaching for effectiveness assessment, like the Simulation Effectiveness Tool-Modified (SET-M) [38, 39]. Objective measures were less commonly incorporated in studies and were mainly focused on areas such as CPR and injection skill training. Sensors were also utilized to collect chest compression (CC) rate, CC depth, needle entry angle or depth to assess the effectiveness of HMD-based AR and MR training [44–46]. Another study used cameras combined with artificial intelligence algorithms to identify metrics, such as the nursing students’ level of engagement when interacting with simulated patients [75]. According to the results of controlled trials, the use of HMD-based AR and MR in nursing education significantly enhanced the knowledge and skill levels of nursing students compared to traditional methods [25, 43]. There is a study compared the effectiveness of using HMD-based AR and MR with conventional methods such as papers in learning anatomy [50]. The results indicated no significant difference between HMD-based AR and MR and other learning methods, but students showed a preference for using AR in anatomy learning.

#### Experiences

The experiences of learners with the application of HMD-based AR and MR in nursing education were mainly obtained through qualitative research methods, such as open-ended questions and interviews [45, 56, 66]. In mixed-methods research, open-ended questionnaires or interviews were used to collect feedback from nursing learners beyond feasibility and other metrics to complement the outcomes of the feasibility assessment [58]. Four qualitative studies mainly explored the needs, cognitions, and experiences of relevant personnel regarding the application of HMD-based AR and MR in nursing education. On the whole, the research subjects have a relatively high acceptance of the application of such technologies [62, 72, 73], but at the same time, their perceptions of its disadvantages (such as low resolution and poor wireless connection quality) are also relatively obvious [64].

### The use of AR and MR concepts

Among included studies, 57% (25/44) reported using AR, 36% (16/44) indicated they were using MR, and the remaining 7% (3/44) did not specify whether their interventions were AR or MR, instead referring to their approach broadly as XR. There were two forms of research utilizing AR concepts. The first was based on the overlay of flat virtual content. This form did not require triggers and allowed learners to simultaneously see virtual content and the real world [78]. The second form was based on the overlay of 3D virtual content. This overlay sometimes required a trigger, and sometimes not. It typically involved a relative relationship between virtual objects and the real environment. However, there was minimal interaction between the two, with the real world mainly providing tactile feedback, and the virtual content offering visual feedback [51]. In studies utilizing the concept of MR, nine studies used the previously mentioned HoloLens and HoloPatient software. However, in one study that used the concept of MR, the intervention adopted resembles the first type of research mentioned earlier that utilized the concept of AR [79]. One of systems displayed camera-captured real-world scenes and virtual objects concurrently in a HMD, and described it using the MR concept [67]. Three studies adopted the XR concept played images or videos in HMDs, which fundamentally shared the same nature with certain studies using HMD-based AR and MR in teaching methods [24, 59, 60].

## Discussion

This scoping review identified a total of 44 studies using HMD-based AR and MR in nursing education, focusing on the description of the current state of application in nursing education. Relevant studies have been conducted primarily in developed countries. The immersive HMDs were the main HMD type, and skill training or simulation education were the main instructional areas. Outcome measures were still focused on feasibility and experience research, with less exploration of educational effectiveness. Additionally, the current use of concepts such as AR and MR in nursing education research remains somewhat ambiguous.

### Insufficient collaboration across institutions and countries

Among the included studies, most of the studies were from developed countries such as the USA and South Korea. This is consistent with existing research [20, 29, 80]. This may be due to the high manufacturing, usage, and maintenance costs of HMD-based AR and MR devices, and the need for high-level interdisciplinary talents for the development of related applications. This may further exacerbate the gap in nursing education between countries with different levels of development. In the future, international cooperation between countries in this field should be promoted to avoid further deepening the digital divide in nursing education [81]. On the other hand, currently, the applications of HMD-based AR and MR are mainly in universities (80%), which is in line with the current evidence [29]. In the future, more collaborations between schools and hospitals should be established, allowing innovative educational approaches developed in academic settings to be extended into clinical environments, thereby facilitating a smoother transition for nursing learners from school to clinical practice.

### Reasonable combinations of HMDs, hardware, and software

A variety of HMD-hardware-software combinations were included in the study, with different configurations of these three elements shaping the diverse application scenarios of HMD-based AR and MR in nursing education. The trade-offs between cost and effectiveness also serve as important references for nursing educators when selecting specific implementation models. Different HMDs vary in terms of simulation fidelity and cost. Other types of hardware can provide feedback that current HMD-based AR and MR systems are unable to offer. Among the three types of software mentioned in the included studies, self-developed software offers full customization to meet the specific needs of various nursing education scenarios. However, it faces problems such as high development costs, and immature user experience. Commercial software is relatively mature. Although it cannot meet personalized needs, it does not require development and has a mature experience. The built-in player of the device does not require any additional cost but the interactivity and immersion that can be achieved are also the lowest. For different HMDs, hardware, and software, nurse researchers and educators should carefully consider their available resources and select the most cost-effective combination based on the educational content and the specific needs of the learners, in accordance with the principle of person-centeredness.

### Different values in skill training and knowledge learning

Currently, the application of HMD-based AR and MR in nursing education mainly focuses on skill training. From a mechanistic perspective, intuitive visual feedback can enhance learners’ confidence and reduce cognitive load and anxiety by providing a simple and enjoyable learning experience [45]. In addition to skills training or simulation education, HMD-based AR and MR were used for knowledge learning, such as anatomical knowledge. Using HMD-based AR and MR for anatomy helps learners to use 3D visualization to learn difficult-to-view anatomical structures, improves spatial awareness, and provides students with the opportunity for repeated review unlike cadaver dissection learning which has a time limit. This is consistent with the results of existing evidence [82, 83]. However, most current HMD-based AR and MR applications are designed for specific scenarios. Although some of these applications incorporate both theoretical instruction and skills training, they lack a systematic design that integrates theory with skill throughout the learning process. The development of nursing competencies, however, requires a deep integration of theoretical knowledge and practical experience. Moreover, in clinical settings, nurses must collaborate with other healthcare professionals to perform a range of tasks. In the future, HMD-based AR and MR nursing education systems should be developed to support continuous, multi-scenario training that tightly integrates theoretical learning with practical application.

### Three-dimensional classification of HMD-based AR and MR

**Fig. 3:**
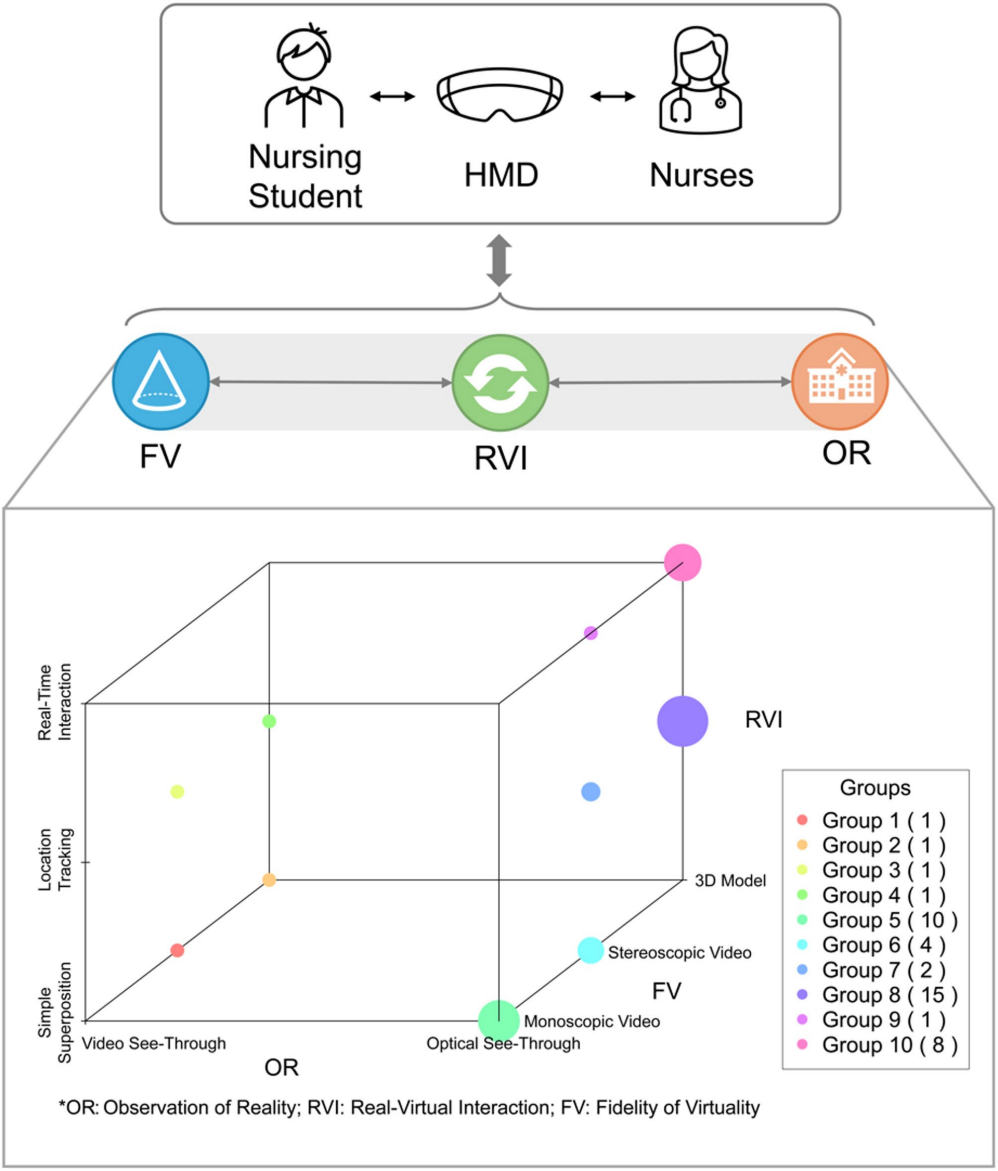
Classification of HMD-based AR and MR used in the included studies

In the use of concepts of AR and MR, there was a lack of standardization and clarity within the literature. Studies utilized different concepts sometimes exhibit similarities in interventional approaches. A more detailed description of this concept should be provided. According to previous research, MR is a broader concept that can be presented through the concept of a “virtuality continuum”. In this continuum, the two poles are AR and augmented virtuality. This study also proposed that MR technology can be divided into three dimensions: Extent of World Knowledge, Reproduction Fidelity, and Extent of Presence Metaphor [84]. Some studies also divide AR and MR technologies from three dimensions: Immersion, Interaction, and Information [85]. Our research is based on previous evidence and combines the characteristics of HMD-based AR and MR applications in nursing education, using the following three elements, including observation of reality (OR), virtual-real interaction (VRI), and fidelity of virtuality (FV), to describe this concept [21, 84, 85]. The description and classification of the three dimensions are shown in Table 2. According to this classification method, HMD-based AR and MR in the literature included in this study can be divided into 10 groups (Fig. 3). In future research, AR or MR should not be simply used to represent interventions. Instead, a detailed description should be made from the above three dimensions, and the influence of different classifications of the three dimensions on the application of this technology in nursing education should be explored in the future.

**Table 2:** Conceptual description of HMD-based AR and MR in nursing education

Dimensions	Description	Classification
Fidelity of Virtuality (FV)	The degree of simulation of virtual objects in the learner’s field of vision.	1. Monoscopic video: Play video on a single channel like stationary monitors. 2. Stereoscopic video: Dual channels are played separately for both eyes, enabling depth perception. 3. 3D model: HMD is capable of rendering high-resolution 3D models.
Real-Virtual Interaction (RVI)	The degree of interaction between virtual and reality in the learner’s field of vision.	1. Simple superposition: Learners can see both virtual and real simultaneously, but without any interaction between the two. 2. Location tracking: HMDs can perceive the real environment and place virtual objects at specific locations in the real environment. 3. Real-time interaction: The device can scan and remodel the real environment, allowing virtual objects to interact with the real environment just like real objects.
Observation of Reality (OR)	The degree of restoration of the real environment in the learner’s field of vision (observation method).	1. Video see-through: HMD reproduces the real scene by capturing the real scene through the camera. 2. Optical see-through: Learners can directly see the real environment through the translucent display.

### Future directions

Although HMD-based AR and MR education has great potential for providing complex and highly accurate nursing education, and simulation-based training for nursing education is rapidly developing, however, there is still a lack of effectiveness assessment of educational outcomes in current research. At this stage, the assessments are still focused on feasibility, which are mostly developed by researchers or borrowed from other digital tools like VR, and lack specific proprietary assessment methods for HMD-based AR and MR [38, 47]. Further comparative studies are still needed in the future to develop assessment methods specific to HMD-based AR and MR, and to compare the effectiveness between different HMD-based AR and MR approaches, HMD-based AR and MR versus traditional methods, as well as between HMD-based AR and MR and VR. In the current market, high-end immersive HMDs are still relatively expensive, user-friendly platforms need to be developed to provide cost-effective alternatives to advanced mediums in the future so that more students can have equitable access to this digital technology [86]. At the same time, the application of HMD-based AR and MR also poses new challenges to the digital literacy of nursing educators and learners, highlighting the need to strengthen competencies in this area [5]. Furthermore, HMD-based AR and MR should be integrated with the latest advancements in artificial intelligence technology, including Large Language Models and Computer Vision. This integration aims to not only enhance the realism of simulation-based teaching but also to empower the assessment of educational outcomes [75, 87].

### Study limitations

This study included only literature in Chinese and English, and did not systematically search for gray literature, which may result in the omission of some evidence and introduce potential bias. On the other hand, due to the intention to provide a comprehensive review of HMD-based AR and MR in the field of nursing education, some specific application areas may not be discussed in sufficient detail; future reviews could focus on these individual domains.

## Conclusions

This scoping review described the status, strengths, and challenges of HMD-based AR and MR use in nursing education, with further definition of AR and MR. HMD-based AR and MR in nursing education were still in the developmental stage, and feasibility studies showed that the use of HMD-based AR and MR in nursing education could be usable in helping learners obtain nursing skills and knowledge. Although the use of HMD-based AR and MR still faces challenges at the current time, they can motivate nursing education. In the use of HMD-based AR and MR in nursing education, there is a need to develop assessment tools specific to the effectiveness of HMD-based AR and MR, and to conduct large-sample randomized controlled trials to evaluate the effectiveness and impact of HMD-based AR and MR in nursing education. Researchers should also provide a standardized description of intervention measures from three perspectives: OR, VRI, and FV. Ultimately, this technology will be used to cultivate better nursing talents, further enhancing human health.

## Electronic supplementary material

Below is the link to the electronic supplementary material.

Supplementary Material 1

Supplementary Material 2

Supplementary Material 3

## Data Availability

The authors confirm that the data supporting the findings of this study are available within the article and its Supplementary materials.

## Abbreviations

XR: Extended reality
VR: Virtual reality
AR: Augmented reality
MR: Mixed reality
HMD: Head-mounted display
OR: Observation of reality
VRI: Virtual-real interaction
FV: Fidelity of virtuality
NASA-TLX: National aeronautics and space administration task load index
CC: Chest compression
CPR: Cardiopulmonary resuscitation
SDS: Simulation design scale
SCLS: Self-confidence in learning scale
IMMS: Instructional materials motivation survey
SSSC: Student satisfaction and self-confidence
ESP: English for specific purpose
PSSUQ: Post-study usability questionnaire
SUS: System usability scale
SET-M: Simulation effectiveness tool-modified
VRSQ: Virtual reality sickness questionnaire
SSES: Satisfaction with simulation experience scale
ECMO: Extracorporeal membrane oxygenation
IPE: Interprofessional education
