# Supplementary material for: Application of head-mounted display-based augmented and mixed reality in nursing education: a scoping review
Source: BMC Nurs. 2025 Sep 2;24:1150. doi: 10.1186/s12912-025-03413-1 (PMC12403271; doi:10.1186/s12912-025-03413-1)
Supplement: Supplementary file 1 — Supplementary Material 1 [file 12912_2025_3413_MOESM1_ESM.docx]

**Search Strategy**

### CNKI

((TKA%=(护理 OR 护士 OR 助产) AND TKA%=(教育 OR 学习 OR 教学 OR 仿真 OR 学生 OR 本科 OR 硕士)) OR TKA%=(护理教育 OR 护理学生 OR 护理教学 OR 护理学习 OR 护生)) AND TKA%=(增强现实 OR 混合现实 OR hololens OR 谷歌眼镜 OR AR眼镜 OR magic leap)

**Fliter:** 同义词扩展

**Hits:** 13

### Wanfang

(((题名或关键词=("护理" OR "护士" OR "助产") AND 题名或关键词=("教育" OR "学习" OR "教学" OR "仿真" OR "学生" OR "本科" OR "硕士")) OR 题名或关键词=("护理教育" OR "护理学生" OR "护理教学" OR "护理学习" OR "护生")) AND 题名或关键词=("增强现实" OR "混合现实" OR "hololens" OR "谷歌眼镜" OR "AR眼镜" OR "magic leap")) OR (((摘要=("护理" OR "护士" OR "助产") AND 摘要=("教育" OR "学习" OR "教学" OR "仿真" OR "学生" OR "本科" OR "硕士")) OR 摘要=("护理教育" OR "护理学生" OR "护理教学" OR "护理学习" OR "护生")) AND 摘要=("增强现实" OR "混合现实" OR "hololens" OR "谷歌眼镜" OR "AR眼镜" OR "magic leap"))

**Fliter:** 中英文扩展, 主题词扩展, 中文

**Hits:** 39

### VIP

(((M=("护理" OR "护士" OR "助产") AND M=("教育" OR "学习" OR "教学" OR "仿真" OR "学生" OR "本科" OR "硕士")) OR M=("护理教育" OR "护理学生" OR "护理教学" OR "护理学习" OR "护生")) AND M=("增强现实" OR "混合现实" OR "AR" OR "MR" OR "hololens" OR "谷歌眼镜" OR "AR眼镜" OR "magic leap")) OR (((R=("护理" OR "护士" OR "助产") AND R=("教育" OR "学习" OR "教学" OR "仿真" OR "学生" OR "本科" OR "硕士")) OR R=("护理教育" OR "护理学生" OR "护理教学" OR "护理学习" OR "护生")) AND R=("增强现实" OR "混合现实" OR "hololens" OR "谷歌眼镜" OR "AR眼镜" OR "magic leap"))

**Filter:** None

**Hits:** 68

### CBM

**#1** "护理"[常用字段: 智能] OR "护士"[常用字段: 智能] OR "助产"[常用字段: 智能]

**#2** "教育"[常用字段: 智能] OR "学习"[常用字段: 智能] OR "教学"[常用字段: 智能] OR "仿真"[常用字段: 智能] OR "学生" [常用字段: 智能] OR "本科"[常用字段: 智能] OR "硕士"[常用字段: 智能]

**#3** #1 AND #2

**#4** "护理教育"[常用字段: 智能] OR "护理学生"[常用字段: 智能] OR "护理教学"[常用字段: 智能] OR "护理学习"[常用字段: 智能] OR "护生"[常用字段: 智能]

**#5** #3 OR #4

**#6** "增强现实"[常用字段: 智能] OR "混合现实"[常用字段: 智能] OR "hololens"[常用字段: 智能] OR "谷歌眼镜"[常用字段: 智能] OR "AR眼镜"[常用字段: 智能] OR "magic leap"[常用字段: 智能]

**#7** #5 AND #6

**Filter:** None

**Hits:** 22

### PubMed

**#1** Nursing[Mesh] OR nursing*[tw] OR Nurses[Mesh] OR nurse*[tw] OR Midwifery[Mesh] OR midwife*[tw] OR midwives*[tw]

**#2** Education[Mesh] OR educat*[tw] OR Learning[Mesh] OR learn*[tw] OR Teaching [Mesh]  OR teach*[tw] OR "Computer Simulation"[Mesh] OR "Simulation Training"[Mesh] OR "simulation base*"[tw] OR simulat*[tw] OR "high fidelity"[tw] OR Students[Mesh] OR student*[tw] OR trainee*[tw] OR apprentice*[tw] OR baccalaureate[tw] OR undergraduate*[tw] OR graduate*[tw] OR post-graduate*[tw] OR pre-licensure[tw] OR pre-registration[tw] OR college*[tw]

**#3** #1 AND #2

**#4** "Education, Nursing"[Mesh] OR "nursing education*"[tw] OR "nurse education*"[tw] OR "Students, Nursing"[Mesh] OR "nursing student*"[tw] OR "pupil nurse*"[tw] OR "nursing learn*"[tw] OR "nursing study*"[tw] OR "nursing teach*"[tw]

**#5** #3 OR #4

**#6** "Augmented Reality"[Mesh] OR "augmented realit*"[tw] OR "mixed realit*"[tw] OR hololens[tw] OR "google glass*"[tw] OR "magic leap"[tw] OR "smart glass"[tw] OR "AR glass"[tw]

**#7** #5 AND #6

**Filter:** None

**Hits:** 130

### Cochrane Library

**#1** MeSH descriptor: [Nursing] explode all trees

**#2** MeSH descriptor: [Nurses] explode all trees

#3 MeSH descriptor: [Midwifery] explode all trees

**#4** (nursing* OR nurse* OR midwife* OR midwives*):ti,ab,kw

**#5** #1 OR #2 OR #3 OR #4

**#6** MeSH descriptor: [Education] explode all trees

**#7** MeSH descriptor: [Learning] explode all trees

**#8** MeSH descriptor: [Teaching] explode all trees

**#9** MeSH descriptor: [Computer Simulation] explode all trees

#10 MeSH descriptor: [Simulation Training] explode all trees

#11 MeSH descriptor: [Students] explode all trees

**#12** (educat* OR learn* OR teach* OR (simulation NEXT base*) OR simulat* OR "high fidelity" OR student* OR trainee* OR apprentice* OR baccalaureate OR undergraduate* OR graduate* OR post-graduate* OR pre-licensure OR pre-registration OR college*):ti,ab,kw

**#13** #6 OR #7 OR #8 OR #9 OR #10 OR #11 OR #12

**#14** #5 AND #13

**#15** MeSH descriptor: [Education, Nursing] explode all trees

**#16** MeSH descriptor: [Students, Nursing] explode all trees

**#17** ((nursing NEXT education*) OR (nurse NEXT education*) OR (nursing NEXT student*) OR (pupil NEXT nurse*) OR (nursing NEXT learn*) OR (nursing NEXT study*) OR (nursing NEXT teach*)):ti,ab,kw

#18 #15 OR #16 OR #17

**#19** #14 OR #18

**#20** MeSH descriptor: [Augmented Reality] explode all trees

**#21** ((augmented NEXT realit*) OR (mixed NEXT realit*) OR hololens OR (google NEXT glass*) OR "magic leap" OR (smart NEXT glass*) OR (AR NEXT glass*)):ti,ab,kw

**#22** #19 AND #21

**Filter:** None

**Hits:** 31

### Embase

**#1** nursing*:ti,ab,kw OR nurse*:ti,ab,kw OR midwife*:ti,ab,kw OR midwives*:ti,ab,kw

**#2** educat*:ti,ab,kw OR learn*:ti,ab,kw OR teach*:ti,ab,kw OR 'computer simulation':ti,ab,kw OR 'simulation training':ti,ab,kw OR 'simulation base*':ti,ab,kw OR simulat*:ti,ab,kw OR 'high fidelity':ti,ab,kw OR student*:ti,ab,kw OR trainee*:ti,ab,kw OR apprentice*:ti,ab,kw AND baccalaureate:ti,ab,kw OR undergraduate*:ti,ab,kw OR graduate*:ti,ab,kw OR 'post graduate*':ti,ab,kw OR 'pre licensure':ti,ab,kw OR 'pre registration':ti,ab,kw OR college*:ti,ab,kw

**#3** #1 AND #2

**#4** 'nursing education*':ti,ab,kw OR 'nurse education*':ti,ab,kw OR 'nursing student*':ti,ab,kw OR 'pupil nurse*':ti,ab,kw OR 'nursing learn*':ti,ab,kw OR 'nursing study*':ti,ab,kw OR 'nursing teach*':ti,ab,kw

**#5** #3 OR #4

**#6** 'augmented realit*':ti,ab,kw OR 'mixed realit*':ti,ab,kw OR hololens:ti,ab,kw OR 'google glass*':ti,ab,kw OR 'magic leap':ti,ab,kw OR 'smart glass':ti,ab,kw OR 'ar glass':ti,ab,kw

**#7** #5 AND #6

**Filter:** None

**Hits:** 53

### CINAHL

**S1** MH(Nursing) OR nursing* OR MH(Nurses) OR nurse* OR MH(Midwifery) OR midwife* OR midwives

**S2** MH(Education) OR MH(Learning) OR educat* OR learn* OR MH(Teaching)  OR teach* OR MH("Computer Simulation") OR MH("Simulation Training") OR "simulation base*" OR simulat* OR "high fidelity" OR MH(Students) OR student* OR trainee* OR apprentice*  OR baccalaureate OR undergraduate* OR graduate* OR post-graduate* OR pre-licensure OR pre-registration OR college*

**S3** S1 AND S2

**S4** MH("Education, Nursing") OR "nursing education*" OR "nurse education*" OR MH("Students, Nursing") OR "nursing student*" OR "pupil nurse*" OR "nursing learn*" OR "nursing study*" OR "nursing teach*"

**S5** S3 OR S4

**S6** MH("Augmented Reality") OR "augmented realit*" OR "mixed realit*" OR hololens OR "google glass*" OR "magic leap" OR "smart glass" OR "AR glass"

**S7** S5 AND S6

**Filter:** None

**Hits:** 94

### Web of Science

**#1** TS=(nursing* OR nurse* OR midwife* OR midwives*)

**#2** TS=(educat* OR learn* OR teach* OR "simulation base*" OR simulat* OR "high fidelity" OR student* OR trainee* OR apprentice* OR baccalaureate OR undergraduate* OR graduate* OR post-graduate* OR pre-licensure OR pre-registration OR college*)

**#3** #1 AND #2

**#4** TS=("nursing education*" OR "nurse education*" OR "nursing student*" OR "pupil nurse*" OR "nursing learn*" OR "nursing study*" OR "nursing teach*")

**#5** #3 OR #4

**#6** TS=("augmented realit*" OR "mixed realit*" OR hololens OR "google glass*" OR "magic leap" OR "smart glass*" OR "AR glass*")

**#7** #5 AND #6

**Filter:** None

**Hits:** 273

### Scopus

**#1** TITLE-ABS-KEY(nursing* OR nurse* OR midwife* OR midwives*)

**#2** TITLE-ABS-KEY(educat* OR educate OR learn* OR teach* OR "simulation base*" OR simulat* OR "high fidelity" OR student* OR trainee* OR apprentice*  OR baccalaureate OR undergraduate* OR graduate* OR post-graduate* OR pre-licensure OR pre-registration OR college*)

**#3** #1 AND #2

**#4** TITLE-ABS-KEY("nursing education*" OR "nurse education*" OR "nursing student*" OR "pupil nurse*" OR "nursing learn*" OR "nursing study*" OR "nursing teach*")

**#5** #3 OR #4

**#6** TITLE-ABS-KEY("augmented realit*" OR "mixed realit*" OR hololens OR "google glass*" OR "magic leap" OR "smart glass" OR "AR glass")

**#7** #5 AND #6

**Filter:** None

**Hits:** 354

### IEEE Xplore

(((nursing OR nurse OR midwife*) AND (education* OR educate OR learn OR teach OR simulat* OR "high fidelity" OR student OR trainee OR apprentice  OR baccalaureate OR undergraduate OR graduate OR post-graduate OR pre-licensure OR pre-registration OR college)) OR("nursing education*" OR "nurse education*" OR "nursing student" OR "pupil nurse" OR "nursing learn" OR "nursing study" OR "nursing teach")) AND ("augmented realit*" OR "mixed realit*" OR hololens OR "google glass" OR "magic leap" OR "smart glass" OR "AR glass")

**Filter:** None

**Hits:** 62

### ACM Digital Library (*The ACM Guide to Computing Literature*)

Abstract:((((nursing* OR nurse* OR midwife* OR midwives) AND (educat* OR learn* OR teach* OR "simulation base*" OR simulat* OR "high fidelity" OR student* OR trainee* OR apprentice* OR baccalaureate OR undergraduate* OR graduate* OR post-graduate* OR pre-licensure OR pre-registration OR college*)) OR("nursing education*" OR "nurse education*" OR "nursing student*" OR "pupil nurse*" OR "nursing learn*" OR "nursing study*" OR "nursing teach*")) AND ("augmented realit*" OR "mixed realit*" OR hololens OR "google glass*" OR "magic leap" OR "smart glass" OR "AR glass")) OR Title:((((nursing* OR nurse* OR midwife* OR midwives) AND (educat* OR learn* OR teach* OR "simulation base*" OR simulat* OR "high fidelity" OR student* OR trainee* OR apprentice* OR baccalaureate OR undergraduate* OR graduate* OR post-graduate* OR pre-licensure OR pre-registration OR college*)) OR("nursing education*" OR "nurse education*" OR "nursing student*" OR "pupil nurse*" OR "nursing learn*" OR "nursing study*" OR "nursing teach*")) AND ("augmented realit*" OR "mixed realit*" OR hololens OR "google glass*" OR "magic leap" OR "smart glass" OR "AR glass"))

**Filter:** None

**Hits: 9**

### Engineering Village (Ei)

**#1** (nursing* OR nurse* OR midwife* OR midwives*) wn KY

**#2** (educat* OR learn* OR teach* OR "simulation base*" OR simulat* OR "high fidelity" OR student* OR trainee* OR apprentice*  OR baccalaureate OR undergraduate* OR graduate* OR post-graduate* OR pre-licensure OR pre-registration OR college*) wn KY

**#3** #1 AND #2

**#4** ("nursing education*" OR "nurse education*" OR "nursing student*" OR "pupil nurse*" OR "nursing learn*" OR "nursing study*" OR "nursing teach*") wn KY

**#5** #3 OR #4

**#6** ("augmented realit*" OR "mixed realit*" OR hololens OR "smart glass" OR "AR glass") wn KY

**#7** #5 AND #6

**Filter:** None

**Hits:** 124
