# Supplementary material for: Application of head-mounted display-based augmented and mixed reality in nursing education: a scoping review
Source: BMC Nurs. 2025 Sep 2;24:1150. doi: 10.1186/s12912-025-03413-1 (PMC12403271; doi:10.1186/s12912-025-03413-1)
Supplement: Supplementary file 2 — Supplementary Material 2 [file 12912_2025_3413_MOESM2_ESM.docx]

**Table 1** Characteristics of subjects and interventions

| **Study** | **Sample** | **Participants type** | **AR/MR concept** | **Intervention** |
| --- | --- | --- | --- | --- |
| **Vaughn, 2016** | Prelicensure second or third semester of an accelerated bachelor of science in nursing: 12 **Total: 12** | Nursing students | AR | HMD: Smart glass-Google Glass Hardwares: N^a^ Software: NA^b^ Trigger: N |
| **Barnett, 2017** | First-year nursing students: 5 **Total: 5** | Nursing students | AR | HMD: Smart glass-Vuzix Wrap 1200DX Hardwares: Manikin, Camera Software: Self-developed Trigger: N |
| **Gruenerbl, 2018** | First-year nurse students: 23 Other volunteer participants: 27 **Total: 50** | Nursing students and others | AR | HMD: Smart glass-Google Glass Hardwares: Manikin, pressure sensor, smart watch Software: Self-developed Trigger: N |
| **Hauze, 2018** | **AR/MR group: 53** Nursing students: 53 **Control group: 54** Nursing students: 54 **Total: 107** | Nursing students | MR | HMD: Immersive HMD-HoloLens Hardwares: N Software: HoloPatient Trigger: NI^c^ |
| **Lai, 2018** | Nursing students: 10 **Total: 10** | Nursing students | MR | HMD: Smart phone-based HMD-Google cardboard Hardwares: Computer, Camera Software: Self-developed Trigger: N |
| **Balian, 2019** | Registered nurse: 34 Physician: 8 Advanced practice registered nurse: 2 Technician: 2 Pharmacy: 2  Other: 3 **Total: 51** | Nurses and others | AR | HMD: Immersive HMD-Hololens Hardwares: N Software: CPReality Trigger: N |
| **Chen, 2019** | Junior nursing students: 48  **Total: 48** | Nursing students | AR | HMD: Smart phone-based HMD-Google cardboard Hardwares: N Software: Self-developed Trigger: N |
| **Hoyt, 2019** | **AR/MR group:**  **Written case study + video group: 54** Nursing students: 54 **AR/MR group 2: 54** Nursing students: 53 **Written case group: 54** Nursing students: 54 **Total: 161** | Nursing students | MR | HMD: Immersive HMD-HoloLens Hardwares: N Software: HoloPatient Trigger: N |
| **Sugiura, 2019** | Nursing students: NI Medical students: NI **Total: 60** | Nursing students and others | AR | HMD: Immersive HMD-Wrap920AR Hardwares: N Software: Self-developed Trigger: N |
| **Frost, 2020** | 3 year Baccalaureate of nursing(2nd year): 96 **Total: 96** | Nursing students | MR | HMD: Immersive HMD-Hololens Hardwares: N Software: HoloPatient Trigger: N |
| **Frost, 2020** | Medical students: 3 Nursing students: 6 Occupational therapy: 2 Dietetics students: 2 **Total: 13** | Nursing students and others | MR | HMD: Immersive HMD-Hololens Hardwares: N Software: HoloPatient Trigger: N |
| **Kim, 2020** | Third-year undergraduage students: 30 **Total: 30** | Nursing students | XR | HMD: Smart glass-Vuzix Blade  Hardwares: N Software: Self-developed Trigger: N |
| **Kopetz, 2020** | Nursing students: 29 **Total: 29** | Nursing students | AR | HMD: Smart glass-Google Glass Hardwares: N Software: Self-developed Trigger: N |
| **Leary, 2020** | **AR/MR group: 50** Advanced practice nurse: 5 Nurse: 35 Other: 6 Physician: 1 Physician assistant: 0 Respiratory therapist: 3 **Control group: 50** Advanced practice nurse: 4 Nurse: 37 Other: 5 Physician: 2 Physician assistant: 2 Respiratory therapist: 2 **Total: 100** | Nurses and others | AR | HMD: Immersive HMD-HoloLens Hardwares: N Software: CPReality Trigger: N |
| **Wunder, 2020** | Student registered nurse anesthetists:32 **Total: 32** | Nurses | MR | HMD: Immersive HMD-Magic Leap One Hardwares: N Software: Self-developed Trigger: N |
| **Anderson, 2021** | Acute care nurse practitioner (AGACNP) students: 8 Faulty, instructors, adjuncts, and other hourly simulation personnel: 4 **Total: 12** | Nursing students and others | AR | HMD: Immersive HMD-HoloLens Hardwares: N Software: Self-developed Trigger: N |
| **Bektic, 2021** | **Non-professional: 4** **Professional: 4** Nursing students: NI Faculty members: NI **Total: 8** | Nursing students and others | AR | HMD: Immersive HMD-Magic Leap One Hardwares: haptic device Geomagic Touch Software: Self-developed Trigger: N |
| **Collins, 2021** | Second-year Bachelor of Nursing (BN) students: 99 **Total: 99** | Nursing students | MR | HMD: Immersive HMD-Hololens Hardwares: N Software: HoloPatient Trigger: N |
| **Dias, 2021** | **direct laryngoscopy (DL) group: 15** Nurse: 15 **indirect video laryngoscopy (IVL) group: 15** Nurse: 15 **AR–assisted video laryngoscopy (ARVL) group: 15** Nurse: 15 **Total: 45** | Nurses | AR | HMD: Smart glass-NI Hardwares: Camera on intubator Software: NA Trigger: NI |
| **Kim, 2021** | Undergraduated nursing students: 30 **Total: 30** | Nursing students | XR | HMD: Smart glass-Vuzix Blade  Hardwares: N Software: Self-developed Trigger: N |
| **Lee, 2021** | Nursing students: 31 **Total: 31** | Nursing students | XR | HMD: Smart glass-Google glass  Hardwares: N Software: Self-developed Trigger: N |
| **Toto, 2021** | **AR/MR group: 25** Nurse: 4 Advanced practice provider: 5 Fellow: 6 Resident: 5 Attending: 5 **Control group: 25** Nurse: 2 Advanced practice provider: 6 Fellow: 5 Resident: 6 Attending: 6 **Total: 50** | Nurses and others | AR | HMD: Immersive HMD-HoloLens Hardwares: Manikin, syringe, potentiometer, computer Software: PediSepsisAR Trigger: N |
| **Yoon, 2021** | Nursing students: 31 **Total: 31** | Nursing students | AR | HMD: Smart glass-Google glass EE2 Hardwares: N Software: Self-developed Trigger: N |
| **Zhang, 2021** | **AR/MR group: 80** Nursing students: 80 **Control group: 80** Nursing students: 80 **Total: 160** | Nursing students | AR | HMD: NI Hardwares: N Software: NI Trigger: NI |
| **Adams, 2022** | Nursing students: 120 **Total: 120** | Nursing students | MR | HMD: Immersive HMD-HoloLens Hardwares: N Software: HoloPatient Trigger: N |
| **Anderson, 2022** | Acute care nurse practitioner (AGACNP) learners: 12 **Total: 12** | Nursing students | AR | HMD: Immersive HMD-HoloLens 2 Hardwares: N Software: AresAR Trigger: N |
| **Heo, 2022** | **AR/MR group: 15** Nurses: 15 **Control group: 15** Nurses: 15 **Total: 30** | Nurses | AR | HMD: Immersive HMD-HoloLens 2 Hardwares: mechanical ventilator Software: Self-developed Trigger: QR code |
| **Kang, 2022** | Medical students: 15 Nursing students: 15 **Total: 30** | Nursing students and others | MR | HMD: Immersive HMD-Hololens 2 Hardwares: N Software: HoloPatient Trigger: N |
| **Kim, 2022** | Nursing student: NI Dental hygiene students: NI **Total: 232** | Nursing students and others | AR | HMD: Immersive HMD-HoloLens Hardwares: N Software: AnatomyX Trigger: N |
| **Menon, 2022** | **AR/MR group: 10** Undergraduate nursing students (1st year): 10 **Control group: 7** Undergraduate nursing students (1st year): 7 **Total: 17** | Nursing students | AR | HMD: Immersive HMD-Magic Leap One Hardwares: N Software: Self-developed Trigger: QR code |
| **Qi, 2022** | Final bachelor nursing students: 20 Nursing education staffs: 8 **Total: 28** | Nursing students and others | AR | HMD: Immersive HMD-Hololens Hardwares: N Software: HoloPatient Trigger: N |
| **Kang, 2023** | **Quantitative study: 62** Nursing students: 62 **Qualitative study: 10** Nursing students: 10 **Total: 72** | Nursing students | MR | HMD: Immersive HMD-HoloLens Hardwares: N Software: HoloPatient Trigger: N |
| **Kim, 2023** | Nursing students: 27 **Total: 27** | Nursing students | AR | HMD: Immersive HMD-Hololens 2 Hardwares: N Software: Self-developed Trigger: N |
| **Kim, 2023** | **Expert group: 9** Nursing students: NI Medical students: NI **Novice group: 11** Nursing students: NI Medical students: NI **Total: 20** | Nursing students and others | MR | HMD: Immersive HMD-HoloLens 2 Hardwares: Geomegic Touch haptic device (right hand), Dexmo haptic glove (left hand) Software: Self-developed Trigger: N |
| **Kleinman, 2023** | **Quantative study: 34** **Group A: 16** nurse: 9 Technician: 7 **Group B: 18** Nurses: 16 Technician: 2 **Qualitative study: 34 Total: 36** | Nursing students and others | AR | HMD: Smart glass-Vuzix M400 Hardwares: Adafruit Inertial Measurement Unit Software: Self-developed Trigger: N |
| **Liu, 2023** | **AR/MR group: 25** Nursing students: 25 **Control group: 24** Nursing students: 24 **Total: 49** | Nursing students | MR | HMD: Immersive HMD-Hololens Hardwares: N Software: NI Trigger: N |
| **Nakazawa, 2023** | **AR/MR group: 21** Nursing students: 21 **Control group: 17** Nursing students: 17 **Total: 38** | Nursing students | AR | HMD: Immersive HMD-Hololens Hardwares: N Software: Self-developed Trigger: N |
| **Son, 2023** | Senior nursing students: 30 **Total: 30** | Nursing students | MR | HMD: Immersive HMD-HoloLens Hardwares: N Software: HoloPatient Trigger: N |
| **Stelter, 2023** | Undergraduated nursing students: NI Dental hygiene students: NI **Total: 205** | Nursing students and others | AR | HMD: Immersive HMD-HoloLens Hardwares: N Software: AnatomyX Trigger: N |
| **Woo, 2023** | Nursing students: 8 **Total: 8** | Nursing students | MR | HMD: Immersive HMD-HoloLens 2 Hardwares: Geomagic Touch haptic device, Sense Glove haptic glove Software: Self-developed Trigger: N |
| **Choi, 2024** | **AR/MR group: 30 Control group: 31 Total: 61** | Nurses | MR | HMD: Immersive HMD-HoloLens 2 Hardwares: Manikin Software: Self-developed Trigger: N |
| **Moon, 2024** | 39 senior nursing students **Total: 39** | Nursing students | MR | HMD: Immersive HMD-HoloLens 2 Hardwares: N Software: Microsoft Dynamic 365 software Trigger: N |
| **Arakida, 2024** | **Quantative study: 53** AR/MR Group: 29 Control Group: 24 **Qualitative study: 38** Students: 29 Faculty: 9 **Total: 62** | Nursing students and others | AR | HMD: Immersive HMD: HTC Vive Hardwares: Barometer, Trachea model, Catheter Software: Self-developed Trigger: N |
| **Yoo, 2024** | Nurses from ICU: 24 **Total: 24** | Nurses | AR | HMD: Immersive HMD-HoloLens 2 Hardwares: N Software: Microsoft Dynamic 365 Trigger: N |

^a^ None

^b^ Not applicable

^c^ No information
